# Association between PM_2.5_ air pollution and social deprivation in Western Pennsylvania

**DOI:** 10.1097/EE9.0000000000000386

**Published:** 2025-04-24

**Authors:** Luke Bryan, Ella M Whitman, Henry Bayly, Philip J Landrigan

**Affiliations:** a Boston College Global Observatory on Planetary Health, Chestnut Hill, Massachusetts; b Department of Biostatistics, Boston University School of Public Health, Boston, Massachusetts; c Centre Scientifique de Monaco, Monaco, Monaco

**Keywords:** Air pollution, Social deprivation, Spatial analysis, Pairwise comparison, Environmental justice

## Abstract

**Introduction:**

Fine particulate matter (PM_2.5_) air pollution is a leading environmental cause of morbidity, premature mortality, and loss of human capital. Western Pennsylvania experiences elevated PM_2.5_ concentrations due to industrial and automotive emissions and a unique geography.

**Objective:**

To assess the relationship between annual mean PM_2.5_ concentration and social deprivation at the census block group level in the eight counties of the Pittsburgh Metropolitan Statistical Area (Allegheny, Armstrong, Beaver, Butler, Fayette, Lawrence, Washington, and Westmoreland).

**Methodology:**

2016 Aerosol Optical Depth modeled PM_2.5_ data were obtained at a 1 × 1 km resolution from the National Aeronautics and Space Administration Socio-Economic Data and Applications Center and spatially joined to the 2,008 census block groups that comprise the eight counties of the Pittsburgh Metropolitan Statistical Area. Using the University of Wisconsin Area Deprivation Index, census block groups were stratified into deciles ranging from 1 (least deprived) to 10 (most deprived). A pairwise comparison was conducted to examine the relationship between annual mean PM_2.5_ estimates and social deprivation within and across deciles.

**Results:**

The average ambient PM_2.5_ concentration for the eight counties was 8.54 ± 0.46 µg/m^3^, with block-group concentrations ranging from 5.59 to 15.90 µg/m^3^. We identified a statistically significant, positive association between PM_2.5_ concentration and social deprivation: group 1, representing the least deprived neighborhoods, had the lowest mean PM_2.5_ concentration of 8.70 µg/m³. In contrast, group 10, representing the most deprived areas, had the highest mean PM_2.5_ concentration of 9.58 µg/m³ and was the only group with a PM_2.5_ concentration significantly higher than all other deciles. The association between PM_2.5_ exposure and social deprivation remained statistically significant, even after applying a false discovery rate correction.

**Conclusion.:**

In Western Pennsylvania, PM_2.5_ exposure is significantly associated with social deprivation. Our results indicate that the relationship between PM_2.5_ and area deprivation in urban US Census block groups was strongest in areas with high levels of deprivation. Future policy interventions should prioritize addressing the unique needs of minority communities that are disproportionately exposed to elevated levels of air pollution.

**What this study adds**

The Pittsburgh Metropolitan Statistical Area, with an estimated population of 2.46 million, experiences elevated fine particulate (PM_2.5_) air pollution levels due to a combination of industrial activity and traffic-related emissions, exacerbated by the region’s distinct topographical features that trap pollutants. Using spatial analysis and pairwise comparisons, this observational investigation quantifies the relationship between satellite-derived PM_2.5_ exposure levels and social deprivation at the census block group level, revealing environmental injustices that are often obscured in aggregate-level analyses.

## Background

Fine particulate matter (PM_2.5_) air pollution is a leading environmental driver of morbidity, premature mortality, and loss of human capital.^1^ PM_2.5_ pollution harms human health at every stage of the life course. Exposure during pregnancy induces oxidative stress and leads to placental insufficiency and intrauterine growth restriction.^2^ These physiological changes result in adverse birth outcomes, including low birth weight, small for gestational age, and premature birth,^2,3^ which have health and societal implications that extend into adulthood and result in a lifelong increase in healthcare utilization. In infants and children, PM_2.5_ exposure contributes to adverse neurodevelopment, demonstrated through a variety of indicators, including structural imaging, intelligence quotient (IQ) testing, and increased risk of autism,^4,5^ as well as adverse respiratory outcomes.^6,7^ In adults, PM_2.5_ exposure is associated with myocardial infarction, stroke, chronic obstructive pulmonary disease, and early neurological decline, as demonstrated by increased rates of dementia,^8,9^ metabolic diseases, and cancer.^10,11^ In 2019, ambient particulate matter air pollution was responsible for an estimated 4.14 million deaths globally,^12^ and the health-related costs of this morbidity and mortality were an estimated $8.1 trillion, equivalent to 6.1% of the global economic output.^13^

PM_2.5_ air pollution is unequally distributed at local, regional, and global levels.^14–17^ In the United States, PM_2.5_ concentrations are disproportionately high in low-income and minoritized communities.^16–18^ These disparities can be traced to historical discriminatory practices such as redlining, segregation, unjust zoning laws, and Not-In-My-Back-Yard (NIMBY) policies for siting pollution sources.^19^

Western Pennsylvania is a metropolitan area with a long history of heavy industry, particularly steel manufacturing and coal mining, and elevated levels of several air pollutants, including PM_2.5_. While the air quality in Pittsburgh has improved dramatically since the mid-nineteenth century, the region continues to struggle with elevated concentrations of PM_2.5_ and other air pollutants and has been ranked as one of the worst cities in the US for air quality, receiving an air quality grade of F in the American Lung Association’s “State of the Air 2023.”^20,21^ Seventy percent of Pittsburgh residents live in areas where average annual PM_2.5_ concentrations exceed 10 µg/m^3^, double the World Health Organization’s health-based guideline level.^22^

Western Pennsylvania faces significant exposure to air pollution due to a combination of industrial activities and traffic-related emissions that are exacerbated by the region’s distinct topographical features. Eight counties comprising the Pittsburgh Metropolitan Statistical Area (MSA) were investigated in this study because they represent communities with diverse socioeconomic backgrounds and levels of urbanization, all of which are impacted by varying sources of air pollution.

For example, the Shell Petrochemical Plant, located approximately 30 miles north of Pittsburgh, processes ethane, used as a plastic feedstock. This process releases hazardous pollutants, including PM_2.5_. Notably, the plant was fined $10 million for air quality violations in May 2023. In the city of Clairton, located in Allegheny County, the US Steel Clairton Plant, also known as the Clairton Coke Works, is the largest manufacturer of coke in North America emitted over 1.1 million pounds of air pollution, placing the 36,000 residents living within three miles of the plant at significant risk of elevated exposure.

In recent years, the Marcellus Shale formation that runs through Western Pennsylvania has experienced a surge in natural gas drilling resulting from advancements in hydraulic fracturing (“fracking”). Though gas combustion produces negligible quantities of PM_2.5_, drill rigs, and construction equipment emit large quantities of diesel exhaust and particulates, volatile organic compounds, silica dust, and nitrogen oxides that contribute to air pollution.^23^

Finally, Pittsburgh—known colloquially as the Steel City^24^—and its surrounding suburbs experience a high volume of traffic-related air pollution (TARP) from vehicular exhaust. Exposures to these airborne pollutants are further exacerbated by the area’s topographical and meteorological features. The confluence of the low-lying river valley of the Appalachian Mountains and frequent temperature inversions restrict the vertical dispersion of airborne pollutants, effectively trapping emissions near the Earth’s surface.

This observational analysis aims to assess the relationship between PM_2.5_ exposure and social deprivation in the eight counties (Allegheny, Armstrong, Beaver, Butler, Fayette, Lawrence, Washington, and Westmoreland)that comprise the Pittsburgh MSA.

## Data and methods

### Demographics and PM_2.5_ exposure estimates

We obtained particulate matter estimates from the 1 × 1 km grid. Annual PM_2.5_ data developed by the National Aeronautics and Space Administration Socio-Economic Data and Applications Center.^25^ We used data from 2016, the most recently available year. We applied these PM_2.5_ estimates to the 2,008 census block groups comprising the eight counties and reduced the set of estimates to those points that fell within a bounding box of 39.7>latitude > 41.3 and −80.55 >longitude > −78.9. We obtained all map boundaries (e.g., census block groups) and demographic data (e.g. population estimates) from the 2021 US Census. We used a geospatial analysis to determine which census block groups contained which Socio-Economic Data and Applications Center PM_2.5_ point estimates. If there were any intersections, the average of the estimates served as the average for the census block group. If there were no intersections, the nearest point served as an estimate for the block group. This analysis assumed planar geography using an NAD83 projection; the most appropriate for the region. All analyses were conducted using R Statistical Software (v4.4.1; R Core Team 2024).

### Social deprivation estimates

We approximated the area deprivation of each neighborhood using the Area Deprivation Index (ADI), developed by the University of Wisconsin-Madison School of Medicine and Public Health by Kind and colleagues.^25^ The ADI is a composite measure of 17 indicators of socioeconomic status across four domains: employment, income, education, and housing quality. It enables neighborhood ranking by socioeconomic disadvantage at the state and national level (Supplemental Table S1; https://links.lww.com/EE/A340). The 2,008 census block groups comprising the eight counties of the Pittsburgh MSA were stratified into deciles using data from the 2021 ADI. While most rankings were integers 1–10, some block groups had exception status codes due to low population, high group quarters population, low data quality, or a combination of the above. Block groups with exception status were excluded from this analysis.

### Spatial analysis

To join estimated air pollution levels with census blocks, we ran a spatial intersection analysis, which produced a list of PM_2.5_ point estimates that intersected with each census block group; 1,455 block groups recorded at least one intersection. We calculated an average of the intersecting estimates for these block groups and used that average as the PM_2.5_ estimate for the block group. For the remaining 553 block groups, the point estimate nearest to the shape’s centroid was used as the PM_2.5_ estimate. We then filtered the block-group PM_2.5_ estimates to remove any census block groups with an ADI exception status; 43 block groups had invalid codes and were excluded from this analysis. We stratified the remaining 1,965 block groups by ADI rank.

### Statistical methods

We first assessed the relationship between average annual PM_2.5_ concentration and ADI via an analysis of variance test. The test revealed that at least two of the ADI groups differed significantly in their PM_2.5_ concentration. To elucidate specific differences between ADI groups, we conducted pairwise t-tests. Due to the presence of heterogeneous variances, we employed Welch’s t-tests, which do not assume equal variances. There were nine tests conducted for each ADI group. To counteract the possibility of finding false positives, we applied a False Discovery Rate (FDR) correction to our *P* values.^26^ All observations were assumed to be independent given the distinct estimates for each census block group due to the granularity of our particulate matter data (1 × 1km).

## Results

### PM_2.5_ estimates and population demographics

The mean PM_2.5_ concentration across all census block groups in the Pittsburgh Metropolitan Area was 9.10 ± 0.05 µg/m^3^, with a range of 5.59–15.90 µg/m^3^. The average ambient PM_2.5_ concentration across the eight counties (Allegheny, Armstrong, Beaver, Butler, Fayette, Lawrence, Washington, and Westmoreland) was slightly lower, 8.54 ± 0.46 µg/m^3^ (Table 1). Of the counties investigated, Allegheny County had the highest average ambient PM_2.5_ concentration of 9.58 µg/m^3^ and contained the largest population (1,233,253 people). The total estimated population of the eight counties is 2,457,000 people.

**Table 1.** PM_2.5_ exposure estimates by County^a^

County	Median PM_2.5_ (µg/m^3^)	MeanPM_2.5_ (µg/m^3^)	Min PM_2.5_ (µg/m^3^)	Max PM_2.5_ (µg/m^3^)	Population estimate
Allegheny	9.657275	9.582399	7.278746	15.90197	1,233,253
Armstrong	8.682075	8.55758	7.650255	10.821752	64,747
Beaver	8.41548	8.371136	7.312333	10.916425	165,677
Butler	8.325675	8.237118	7.488829	9.885844	197,300
Fayette	7.985155	8.162996	5.591949	9.533365	125,755
Lawrence	8.25077	8.268628	7.673194	8.988845	84,849
Washington	8.101392	7.965162	6.737478	10.044661	210,383
Westmoreland	8.887499	8.835712	6.836356	10.699734	352,057

a All PM_2.5_ estimates from 2016 and demographic data from 2021.

### General trends between ADI and PM2.5

The PM_2.5_ variances between ADI decile groups were found to be unequal via a Levene test (P = 0.02). Therefore, a Welch analysis of variance test was utilized to assess differences among means, and the results were found to be significant (*P* = 2.2E-16). Table 2 presents the mean PM_2.5_ concentrations for each ADI group.

**Table 2.** PM_2.5_ exposure estimates by ADI groups

ADI state rank	Median PM_2.5_ (µg/m^3^)	Mean PM_2.5_ (µg/m^3^)	Confidence interval	Degrees of freedom	Standard error (SE)
1	8.51414	8.70181	(8.526417, 8.877197)	107	0.089485
2	8.66551	8.80541	(8.643949, 8.966877)	157	0.0823795
3	8.6637	8.86213	(8.713419, 9.010835)	159	0.0758715
4	8.75361	8.98898	(8.801859, 9.176101)	159	0.09547
5	8.77145	8.97211	(8.829103, 9.115119)	219	0.072963
6	8.73301	8.92193	(8.779115, 9.064738)	207	0.072863
7	9.01449	9.12002	(8.958674, 9.281357)	230	0.082317
8	9.16128	9.17297	(9.039847, 9.306093)	235	0.06792
9	9.25833	9.21781	(9.084789, 9.350837)	237	0.067869
10	9.57538	9.58265	(9.463427, 9.701864)	245	0.060826

**Figure 1.**
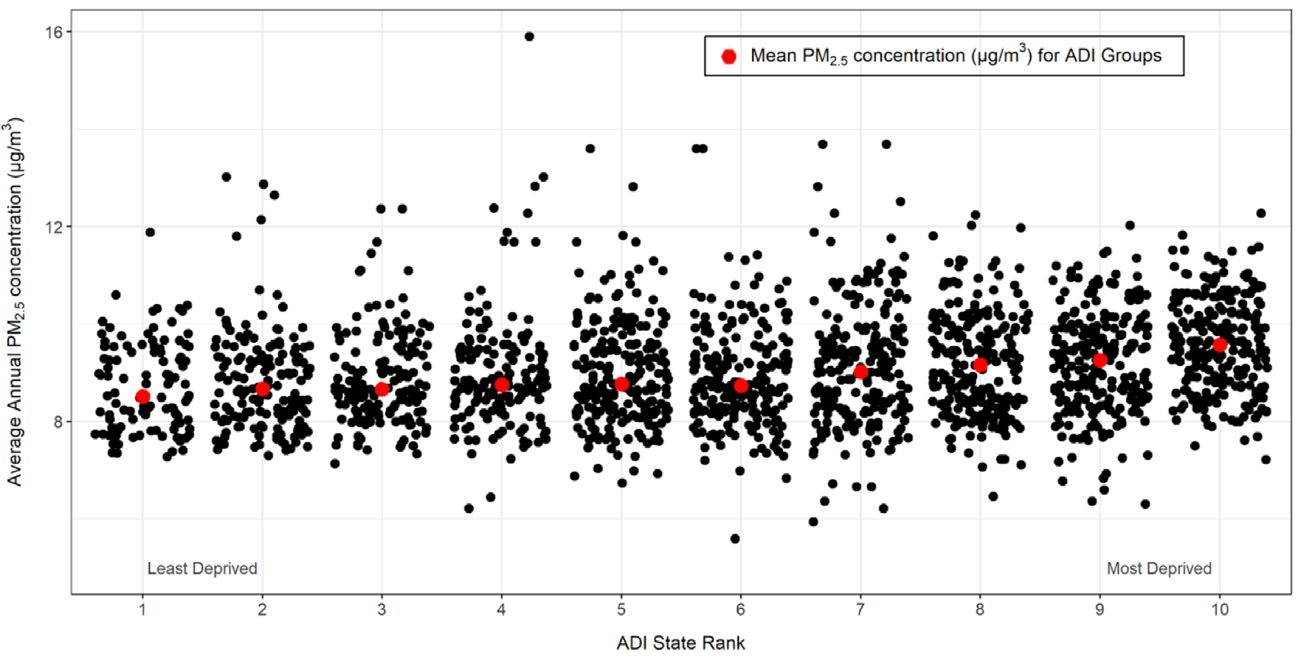
Average annual PM_2.5_ concentrations in Pittsburgh metro area grouped by ADI state rank.

**Figure 2.**
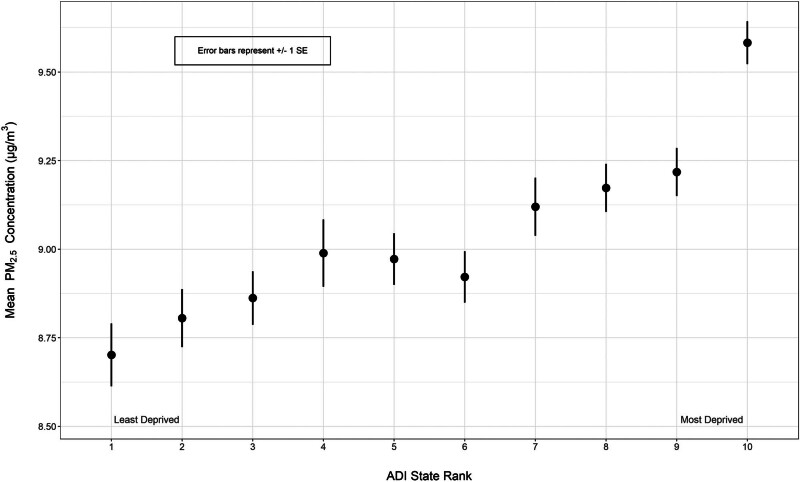
Average annual PM_2.5_ estimates grouped by ADI state rank (vertical bars represent ± 1 standard error).

Notably, group 1, representing the neighborhoods ranked in the lowest decile of deprivation, exhibited the lowest mean PM_2.5_ concentration of 8.70 µg/m³. In contrast, group 10, representing neighborhoods ranked in the highest decile of deprivation, had the highest average PM_2.5_ concentration of 9.58 µg/m³ (Figures 1 and 2). This finding supports the need for post-hoc testing to identify differences in average annual PM_2.5_ concentrations between individual ADI rankings.

### Pairwise comparisons and statistical significance

To further elucidate the relationship between PM_2.5_ and ADI, we conducted pairwise comparisons of average PM_2.5_ concentrations among the ADI groups. Importantly, we compared each ADI rank using unpooled Welch T-tests and used an FDR correction to control the family-wise error rate. The results of the pairwise analyses are summarized in Table 3. Of the 45 pairwise comparisons performed, 24 were initially found to be significant, and 22 were significant after applying the FDR correction.

**Table 3.** Pairwise *P* values with FDR correction of average PM_2.5_ concentration between ADI rankings

ADI Rank	1	2	3	4	5	6	7	8	9	10
1	*NA*	*NA*	*NA*	*NA*	*NA*	*NA*	*NA*	*NA*	*NA*	*NA*
2	0.463	*NA*	*NA*	*NA*	*NA*	*NA*	*NA*	*NA*	*NA*	*NA*
3	0.245	0.654	*NA*	*NA*	*NA*	*NA*	*NA*	*NA*	*NA*	*NA*
4	^a^0.054	0.215	0.37	*NA*	*NA*	*NA*	*NA*	*NA*	*NA*	*NA*
5	^b^0.041	0.199	0.37	0.888	*NA*	*NA*	*NA*	*NA*	*NA*	*NA*
6	0.096	0.37	0.646	0.646	0.654	*NA*	*NA*	*NA*	*NA*	*NA*
7	^b^0.002	^b^0.017	^b^0.043	0.37	0.249	0.118	*NA*	*NA*	*NA*	*NA*
8	^b^1.50 × 10-4	^b^0.002	^b^0.006	0.185	^a^ 0.081	^b^0.028	0.654	*NA*	*NA*	*NA*
9	^b^3.81 × 10-5	^b^4.53 × 10-4	^b^0.002	0.09	^b^0.03	^b^0.008	0.435	0.654	*NA*	*NA*
10	^b^9.50 × 10-13	^b^5.85 × 10-12	^b^1.11 × 10-11	^b^1.92 × 10-6	^b^2.48 × 10-9	^b^1.14 × 10-10	^b^3.98 × 10-5	^b^3.98 × 10-5	^b^2.73 × 10-4	*NA*

a Significant with no correction applied.

b Significant with FDR correction.

Notably, the adjusted *P* value for the comparison between PM_2.5_ exposure in group 10 and all other ADI groups remained statistically significant, even after FDR correction. This finding suggests that the most deprived neighborhoods experience higher levels of PM_2.5_ compared to “low” and “moderately deprived” neighborhoods (e.g., groups 1–9). In contrast, neighborhoods with median levels of deprivation (e.g., group 5) show significant differences in PM_2.5_ exposure only when compared with those in the very lowest (group 1) and highest deprivation categories (groups 8–10), suggesting the relationship between PM_2.5_ and area deprivation in the Pittsburgh MSE is most disparate in deciles that are highest in the ADI.

### Mapping PM2.5 and ADI

Following formal analyses between PM_2.5_ and ADI, we visualized the data in a map format to illustrate geospatial trends that exist in the data for the study area.

**Figure 3.**
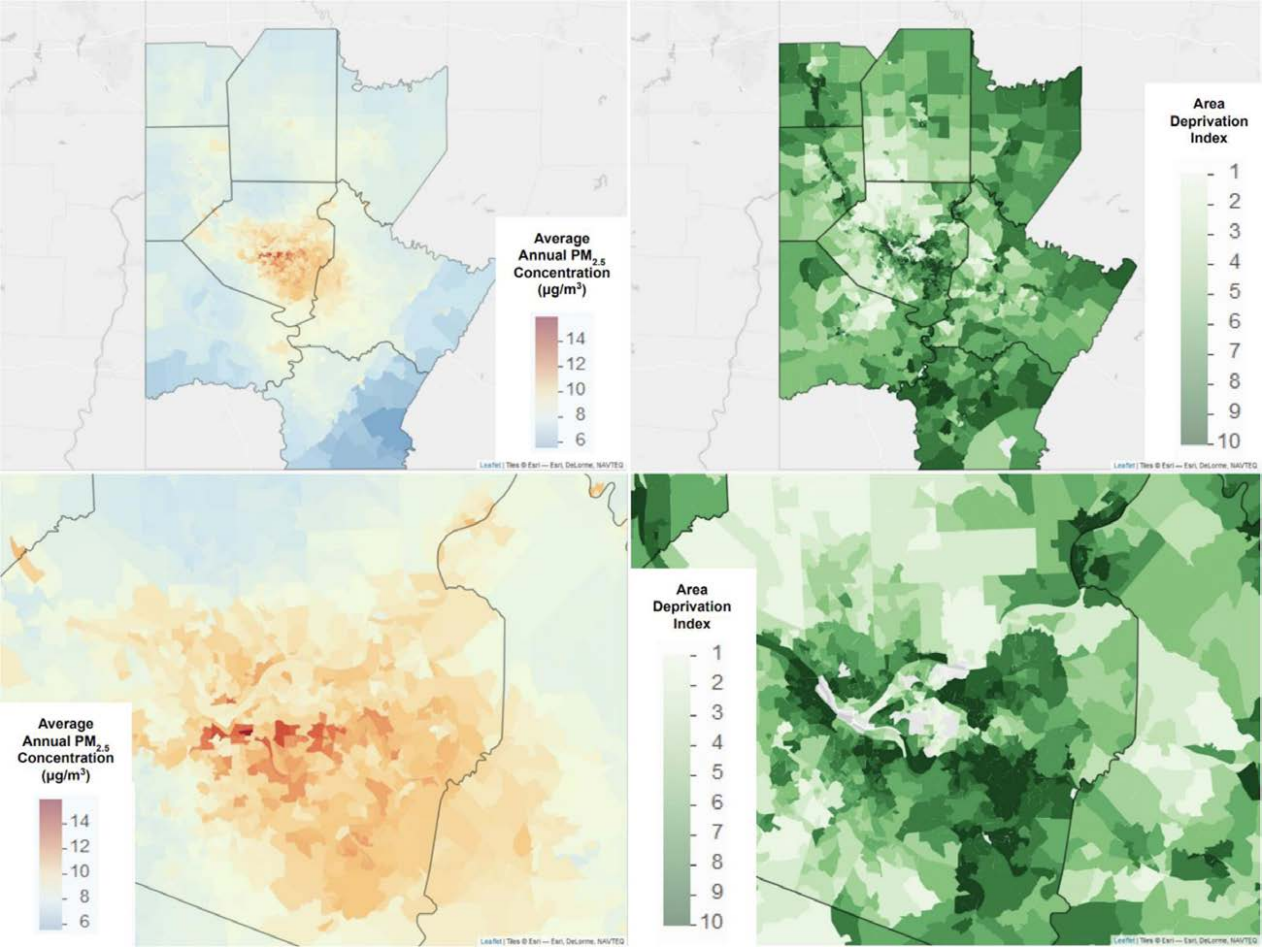
Maps of average annual PM_2.5_ concentrations (2016, in μg/m^3^) and ADI for all census block groups in the Pittsburgh Metropolitan Statistical Area.

Figure 3 shows the annual PM_2.5_ concentrations and ADI for all census block groups. Both maps are color-coded and display the full range of values (5.9–15.9 μg/m^3^, ADI 1–10). PM_2.5_ concentrations are available for all census block groups, while the ADI map excludes census block groups with exclusion codes that were not included in the analysis and do not have an ADI score available. There are a few examples of these block groups along the river in downtown Pittsburgh.

Notably, PM_2.5_ concentrations in the middle of Allegheny County—downtown Pittsburgh—are higher than anywhere else in the state. While most surrounding areas have elevated PM_2.5_ levels, the trend is clearly present on the southeast side of the city, where block groups consistently have exposure concentrations higher than the current US Environmental Protection Agency (EPA) health-based standard (9 μg/m^3^). Furthermore, this region has a high proportion of block groups ranked in the highest ADI deciles (9–10). Conversely, the northern and southwestern sides of the city have notably lower PM_2.5_ concentrations and more census block groups ranked in the lowest ADI deciles (1–2).

## Discussion

Our findings in this study of the relationship between PM_2.5_ air pollution and social deprivation in the Pittsburgh MSA using the Wisconsin ADI show a significant association between air pollution and social deprivation, with the strongest relationship seen in block groups ranked highest in the highest decile of the ADI.

The marked escalation of pollution exposure observed in the most highly deprived populations raises the possibility that groups with higher deprivation are more likely to differ from each other in mean PM_2.5_ concentrations than ADI groups that are not as deprived. In our study, census block groups ranked in the top decile of deprivation by the ADI had PM_2.5_ concentrations significantly higher than all other ADI groups. Simultaneously, there were no significant relationships between any two groups between ADI deciles 1–3 nor in the 2–6 ADI decile range. Since we did not identify significant differences in PM_2.5_ concentration between all adjacent ADI groups, we cannot identify the exact functional form of the relationship between ADI and PM_2.5_ concentration. However, given that we see more significant differences in the more deprived adjacent groups than in the less deprived adjacent groups, our findings suggest that mean PM_2.5_ concentration increases greater in magnitude between deciles of higher deprivation compared with lower.

Another point of discussion is the monotonicity of our findings. Of 45 pairwise comparisons conducted, 22 were significant, and 20 remained significant following the FDR correction. In all of these significant results, the more disadvantaged ADI group had a higher PM_2.5_ concentration. Thus, our significant findings are highly monotonic and suggest that increasing socio-economic disadvantage is associated with exposure to higher annual concentrations of PM_2.5_.

Contravening that explanation, however, is our finding that the most polluted census-block groups were not necessarily in the most deprived groups. Thus, of the 10 census block groups with the highest PM_2.5_ concentrations, all had ADI in the 2–7 range, and of the 20 most polluted block groups 19 were in the 2–7 ADI range. This is despite the fact that ADI group 10 had PM_2.5_ concentrations significantly higher than all other groups and that both the 8 and 9 ADI groups had significantly higher PM_2.5_ concentrations than groups 2, 3, 5, and 6. This finding adds further nuance to the relationship between PM_2.5_ and ADI and challenges the earlier notion that the relationship may be monotonic. Other published studies also indicate nonlinear and nonmonotonic relationships between PM_2.5_ and pollution in urban environments.^27,28^ These findings highlight the importance of using highly granular data in studies of this relationship.

A nationwide survey conducted in China in 2014 by Jiao et al.^27^ found a nonlinear relationship between community socioeconomic status (SES) and community air pollution, with the highest level of air pollution observed in the communities with moderate SES. Additionally, the authors found that the health effects associated with air pollution in different SES groups were not equal. In a study by Bevan et al.^29^, social deprivation and PM_2.5_ exposures were independently associated with county-level age-adjusted cardiovascular mortality, with the relationship between PM_2.5_ and cardiovascular mortality strongest in communities with higher deprivation. Given this important nuance, the authors conclude that interventions to reduce PM_2.5_ exposure may be most impactful in communities with low SES.

The nuanced relationship between PM_2.5_ and ADI that we observed has important implications for public health research. First, it underscores the need to incorporate social determinants of health into studies examining environmental exposures and, by the opposite token, to consider environmental factors in studies of social determinants. Future research should prioritize comprehensive confounder adjustment when designing statistical models, including variables such as socioeconomic status, access to healthcare, and demographic factors. This approach will help clarify complex relationships that might otherwise go unnoticed, reducing the risk of false attribution within causal frameworks. Such analyses will enable the design of targeted public health interventions and policy decisions that effectively mitigate the effects of air pollution on the health of vulnerable populations.

Our findings also underscore the critical importance of examining long-standing inequalities through the lens of environmental justice as opposed to relying solely on air quality measurements. Given the granularity of the disparities we observed, we conclude that community input is critical to advancing environmental justice. It is essential to work directly with communities experiencing high levels of deprivation when designing interventions to mitigate exposure and build resilience.

Additionally, our findings are consistent with the growing body of literature documenting the many ways in which socioeconomic conditions drive intergenerational inequalities resulting from air pollution exposure. For example, using data from 63,165 US census tracts (86% of all census tracts in the United States), Dominici and colleagues found that census tracts with a 1 μg/m^3^ higher PM_2.5_ concentrations in 1982 are associated with statistically significant lower absolute upward mobility in 2015.^30^ This finding helps explain why the most disadvantaged census block groups are disproportionately impacted compared with moderately disadvantaged groups, and why marked disparities persist even after state-wide improvements.

Similar analyses conducted at the national and international levels have found positive relationships between area disadvantage and PM_2.5_ exposure that persist over time and space. In a Texas birth cohort examining seven million pregnant people over the span of 20 years, found that traffic-related air pollution exposure decreased across all participants, but that relative disparities for persistently marginalized groups remained.^31^ In Hong Kong, a cross-sectional analysis identified a significant, positive relationship between ambient PM_2.5_ concentration and increased Social Deprivation Index, a composite indicator comprising socioeconomic status variables (i.e., income, education, and occupation).^28^

Our study has important strengths. We analyzed the relationship between PM_2.5_ estimates using high-resolution 1 × 1 km estimates based on satellite imagery with ADI data at the census block group level, the finest level of detail publicly shared by the Census Bureau. We employed the widely used University of Wisconsin ADI.^26^

Our study also has several limitations. First, though highly localized, our 1 × 1 km estimates assume equal contributions from all grids, which could introduce potential errors in exposure measurements. In reality, air pollution levels can vary significantly within a given grid due to factors such as proximity to industrial sources, roadways, or natural features that influence the dispersion of pollutants. Pollution estimates were generated from 2016, the most recent available dataset, while census block group data from the ADI were taken from 2021. Therefore, our analysis assumes that PM_2.5_ exposure levels remained consistent from 2016 to 2021. This temporal mismatch may have introduced inaccuracies in the validity of our conclusions if significant changes occurred in air pollution exposure or the socio-economic conditions of neighborhoods during that time. Despite this, the use of the most recent available data for both pollution estimates and socio-economic indicators provides a reasonable approximation of exposure levels. Future research would benefit from using temporally aligned data to strengthen the accuracy of findings.

Second, the ADI is a static measure based on specific variables, which may not capture all indicators of deprivation. Also, census block groups were ranked into broad categories of the ADI (e.g., 1–10), which may not reflect the heterogeneity and diversity of individual residents’ experiences, leading to overgeneralization. Additionally, certain census block groups were identified as exclusion groups according to the ADI and thus excluded from this analysis.

Finally, PM_2.5_ exposure estimates may be less accurate in rural areas due to lower spatial resolution in the data these estimates were trained on. Furthermore, satellite-derived air quality data relies on a limited number of monitoring stations, primarily located in urban areas with high population densities.

Future analyses should seek to increase the precision and accuracy of measures of both air pollution and social deprivation. For example, conducting subset analyses that quantify individual-level data via personal monitoring devices to capture key variations that could generate new insights concerning specific drivers of health disparities. By integrating these data with continuous (noncategorical) socio-economic variables and select demographic factors, researchers can better understand how individual behaviors, environmental contexts, and community characteristics influence exposure levels.

Additionally, examining intersectionality—how overlapping identities such as race, gender, and socioeconomic status interact to affect exposure and health outcomes—will provide a more nuanced understanding of vulnerability. Black, Indigenous, and People of Color (BIPOC) often have lower income, education, and employment levels compared with their white peers.^32–34^ Since ADI does not factor in race and ethnicity, BIPOC communities may experience compounding or even synergistic effects of pollution and social deprivation due to the combination of environmental racism with the negative trends found in this study. This comprehensive approach will enable more tailored public health interventions and inform policy decisions aimed at reducing air pollution impacts on diverse and marginalized populations.

## Conclusion

In this investigation of the association between PM_2.5_ air pollution levels and social deprivation at the census block level across eight counties in the Pittsburgh MSA, we found that PM_2.5_ is significantly associated with deprivation. The association between PM_2.5_ and ADI was most disparate among the most highly disadvantaged groups, and our study highlighted the granularity at which air pollution disparities can exist. Policy actions to mitigate PM_2.5_ exposure must consider the impact, distribution, and needs of highly disadvantaged communities to design targeted interventions that effectively advance environmental justice. Future research investigating the relationship between air pollution and health should carefully consider social disadvantage factors when designing statistical models.

## Conflicts of interest statement

The authors declare that they have no conflicts of interest with regard to the content of this report.

## ACKNOWLEDGMENTS

*The authors sincerely thank the Heinz Endowments for its generous support and Dr. Amy Kind, MD, PhD, developer of the Area Deprivation Index (ADI).*

## Supplementary Material


