# Supplementary material for: Association between PM2.5 air pollution and social deprivation in Western Pennsylvania
Source: Environ Epidemiol. 2025 Apr 24;9(3):e386. doi: 10.1097/EE9.0000000000000386 (PMC12026379; doi:10.1097/EE9.0000000000000386)
Supplement: Supplementary file 1 [file ee9-9-e386-s001.pdf]

Supplemental Table S1: Census Variables in the Area Deprivation Index

Domain	Variable
Education	% Population aged 25 years or older with less than 9 years of education
	% Population aged 25 years or older with at least a high school diploma
	% Employed population aged 16 years or older in white-collar occupations
Income/employment	Median family income in US dollars
	Income disparity
	% Families below federal poverty level
	% Population below 150% of federal poverty level
	% Civilian labor force population aged 16 years and older who are unemployed
Housing	Median home value in US dollars
	Median gross rent in US dollars
	Median monthly mortgage in US dollars
	% Owner-occupied housing units
	% Occupied housing units without complete plumbing
Household characteristics	% Single-parent households with children younger than 18
	% Households without a motor vehicle
	% Households without a telephone
	% Households with more than 1 person per room

As described by Kind AJH, Buckingham W. Making Neighborhood Disadvantage Metrics Accessible: The Neighborhood Atlas. N Engl J Med. doi:10.1056/NEJMp1802313
